# Changes in Antibiotic Susceptibility of *Helicobacter pylori* in the Course of Eight Years in the Zaanstreek Region in The Netherlands

**DOI:** 10.1155/2013/625937

**Published:** 2013-03-12

**Authors:** R. J. L. F. Loffeld, B. F. M. Werdmuller

**Affiliations:** ^1^Department of Internal Medicine, Zaans Medisch Centrum, Zaandam, The Netherlands; ^2^Department of Microbiology, Zaans Medisch Centrum, Zaandam, The Netherlands

## Abstract

*Background*. Failure of anti-*Helicobacter* therapy is the result noncompliance and resistance to the prescribed antibiotics. *Aim*. Antibiotic susceptibility of *H. pylori* was determined in native Dutch patients and patients of Turkish descent. *Methods*. In a period of eight years a total of 925 strains of *H. pylori* were cultured. Bacterial susceptibility was successfully determined in 746 (80.6%) of these isolates. Three hundred and nine strains (33%) originated from patients of Turkish descent. *Results*. In total clarithromycin resistance was found in 146 (20.5%) strains, metronidazole resistance in 147 (19.9%) strains. Amoxicillin resistance was found to be present in five strains. There is a slight but nonsignificant decrease in the percentage of clarithromycin-resistant strains in the consecutive period of eight years from 20% to 18%. No changes were seen in the consecutive years in metronidazole resistance. The number of clarithromycin-resistant strains decreased in Turkish patients, not in native Dutch patients. *Conclusion*. Resistance did not change significantly in consecutive years. But clinicians should take not only the antibiotic history into account but also ethnicity before prescribing metronidazole or clarithromycin.

## 1. Introduction

The discovery of *H. pylori* and the recognition of its clinical importance have been a major breakthrough in clinical medicine. Eradication of the bacterium definitely cures peptic ulcer disease. Several treatment regimens, mostly consisting of acid-suppressive drugs in combination with two antibiotics, have been applied with different success rates. Failure of therapy is the result of not only noncompliance but also of resistance of the bacterium to the prescribed antibiotics. Success of anti-*Helicobacter* therapy substantially decreases in the presence of a resistant strain [1]. 

In a previous study from our group it was shown that the resistance of *H. pylori* to clarithromycin rose in consecutive years while resistance to metronidazole showed a slight decrease [2]. Moreover, if ethnicity of the patients was taken into account, than it was shown that the patients other than native Dutch patients had a higher percentage of strains resistant to clarithromycin and metronidazole.

It is interesting to investigate further development of bacterial resistance. The present study was performed in order to evaluate changes in bacterial resistance in a more recent time period.

## 2. Material and Methods

Patients undergoing upper gastrointestinal endoscopy, in the Zaans Medisch Centrum, the community hospital of the Zaanstreek region in The Netherlands, were included. The reason for doing the endoscopy, obviously, is presence of upper abdominal complaints, anemia, or reflux complaints. Biopsy specimens from the gastric antrum for detection of *H. pylori* were taken when clinically indicated by the endoscopist. In addition, cultures from two hospitals in the vicinity were included as well. The biopsy specimens were processed at the laboratory for microbiology in the Zaans Medisch Centrum. This laboratory has a regional function and serves three hospitals.

From all patients demographic data and ethnicity were collected.

In the present study all cultures from January 2004 until December 2011 were included. Only the first culture in a patient was included. Cultures taken because of followup after prior attempts to eradicate *H. pylori* were excluded.

A large group of people from Turkish descent (immigrants from Turkey) lives in the Zaanstreek region. This group was studied separately for presence of antibiotic resistance.

The biopsy specimens taken during endoscopy were transported in sterile physiologic saline to the laboratory as soon as possible (usually within two hours).

The specimens were inoculated on Skirrow's medium, containing vancomycin, trimethoprim lactate, and amphotericin B. The plates were incubated at 37°C in a humid microaerophilic environment during at least 7 days. Microaerophilic conditions were maintained with the Anoxomat Mark III (Mark Microbiology). As a control a *Campylobacter* strain was taken. Growth was determined at three to four and seven to eight days. Suspected colonies were Gram stained and evaluated by testing catalase, oxidase, and urease production [3]. Antibiotic susceptibility of *H. pylori* was determined by *E*-test (Biomerieux) on Columbia agar (Oxoid, Basingstoke, United Kingdom) enriched with 7% sheep blood [4]. 

*E*-test strains were inoculated in McFarland 3 turbidity. Incubation was done under microaerophilic conditions at 35°C for 3-4 days conforming to *E*-test guidelines (Biomeriuex). Interpretation for the susceptibility used EUCAST breakpoints for metronidazole at MIC 8.0 mg/L and clarithromycin at MIC SC 0.25 mg/L. Since 2007 susceptibility to amoxicillin was also routinely tested with a MIC SC of 0.125 mg/L.

Statistical analysis was done with chi-square test for contingency tables. A value below 0.05 was considered statistically significant.

## 3. Results

In the period of eight years a total of 925 strains of *H. pylori* were cultured (469 men, 51%, and 456 women, 49%). Bacterial susceptibility was successfully determined in 746 (80.6%) of these isolates. In the remainder the strains were not viable after initial culture. A total of 146 cultures originated from the two other hospitals. The endoscopic diagnosis in these patients was not available. The diagnosis in Table 1 represents the macroscopic endoscopic findings seen in patients undergoing endoscopy in the Zaans Medisch Centrum.

Three hundred and nine strains (33%) originated from patients of Turkish descent.

Clarithromycin resistance was found in 146 (20.5%) strains, metronidazole resistance in 147 (19.9%) strains. In addition, amoxicillin resistance was found to be present in five strains. Five out of 477 cultures (1%) tested positive for resistance.  Table 2 shows the resistance pattern of *H. pylori* strains. In Table 3 the resistant strains in Dutch patients and Turkish patients are shown in the consecutive years.

**Figure 1:**
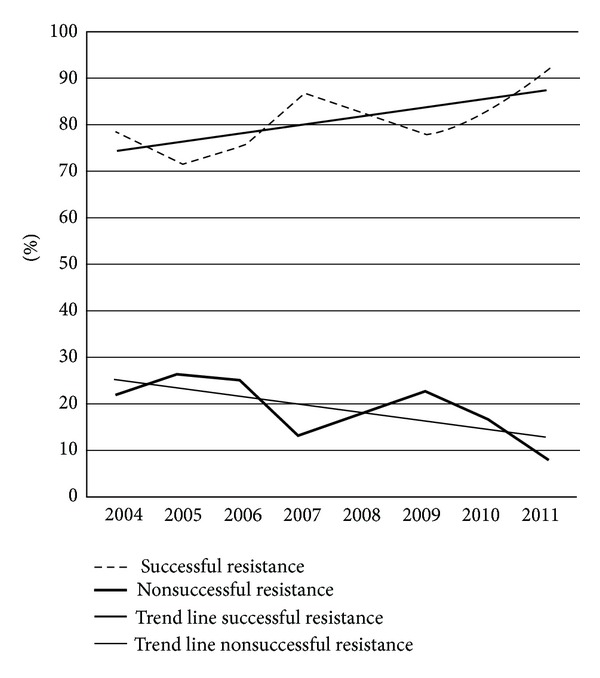
The percentage and trend lines of successful and nonsuccessful determination of antimicrobial susceptibility in the consecutive eight years.

Figure 1 shows the result of determination of bacterial resistance in the consecutive years. As can be seen the numbers of successful determination of antibiotic resistance gradually increased.

**Figure 2:**
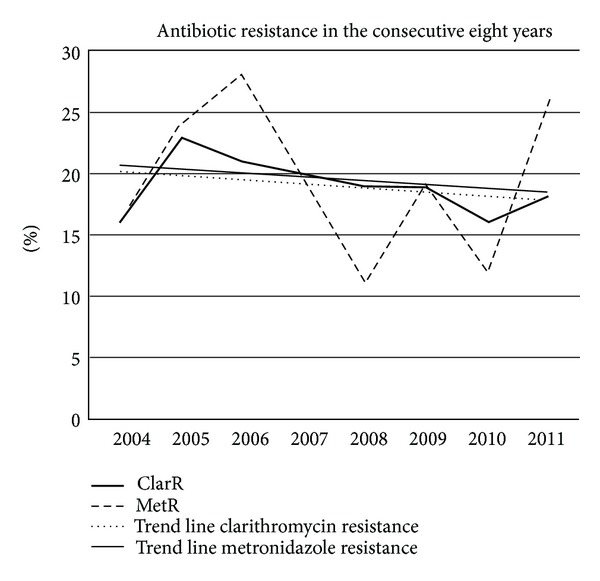
The percentage of susceptible and nonsusceptible strains in the consecutive eight years. ClarR: resistance for clarithromycin, MetR: resistance for metronidazole.

Figure 2 shows the percentage of clarithromycin- and metronidazole-resistant strains. Although there is a yearly fluctuation, trend lines show a slight decrease in the percentage of resistant strain in the consecutive years from 20% to 18%. However, this did not reach statistical significance. Only for metronidazole a trend was noted (*P* = 0.06).

**Figure 3:**
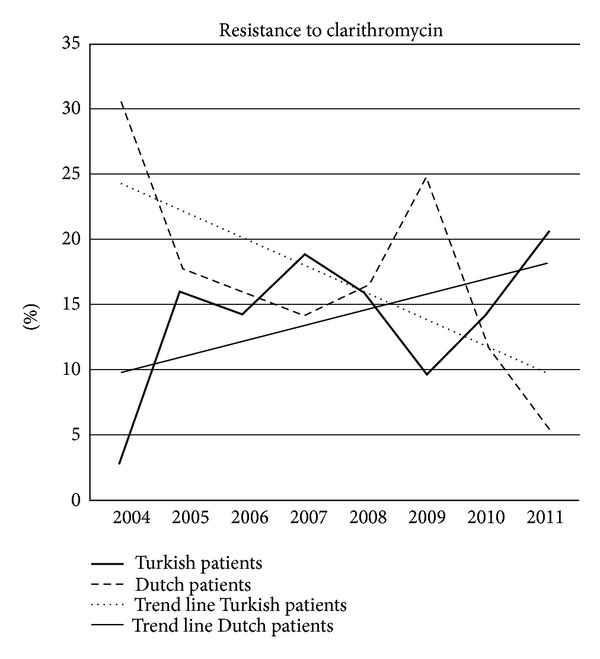
Resistance to clarithromycin in Dutch patients and Turkish patients in the consecutive eight years.

**Figure 4:**
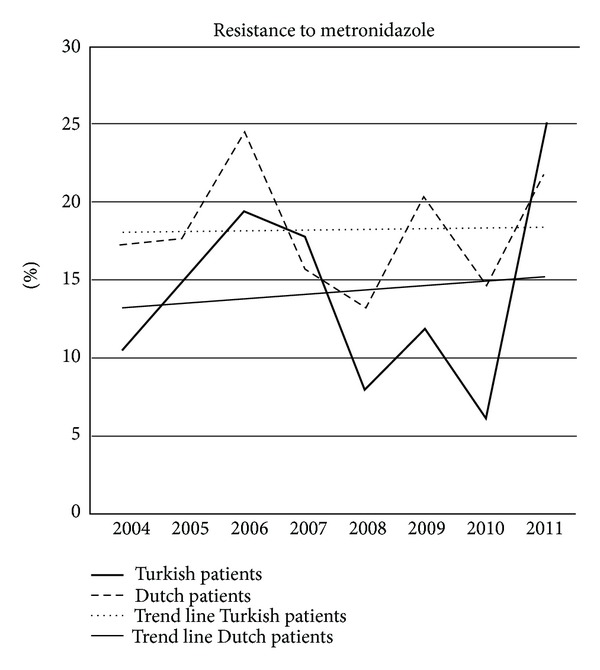
Resistance to metronidazole in Dutch patients and Turkish patients in the consecutive eight years.

Figure 3 shows the clarithromycin resistance in native Dutch patients and patients from Turkish descent in the consecutive years. The trend lines show a statistically significant decrease of resistance in the Turkish patients (*P* = 0.01) while resistance increased in the native Dutch patient group.  Figure 4 shows the same for metronidazole resistance. No significant changes were seen in the consecutive years.

## 4. Discussion

The resistance of *H. pylori* to antibiotics is the main factor affecting the efficacy of the current regimens. The mechanisms of resistance to clarithromycin, metronidazole, quinolones, amoxicillin, and tetracycline are known in detail (point mutations, redox intracellular potential, pump efflux systems, and membrane permeability) [5]. 

A proton-pump inhibitor (PPI), clarithromycin-based, triple therapy has been the mainstay of treatment for *H. pylori* eradication in the past 15 years. Due to a steady increase in *H. pylori* resistance, this triple clarithromycin-based treatment has become less efficacious. The rate of eradication of *H. pylori* with standard triple therapy using omeprazole, amoxicillin, and clarithromycin (OAC) has become unacceptably low in populations with high rates of clarithromycin resistance [6]. On the other hand, the eradication rate of *H. pylori* after this triple therapy for up to 14 days still is effective in other areas. In a large meta-analysis from Spain a mean *H. pylori* cure rate of 80% (95% CI = 77–82%) by intention to treat and 83% (81–86%) by per protocol was shown. However, mean clarithromycin resistance rate was rather low, 8% (5–10%) [7]. 

In the course of the years the number of strains successfully cultured for testing of resistance gradually increased from 79% to over 90%. The reason for this is not obvious. There was no change in handling or culture technique of the biopsy specimens. Perhaps it shows the growing experience in the laboratory with cultures of *H. pylori*.

In comparison with the earlier study in our population metronidazole resistance decreased from 25.8% to 19.9%, while clarithromycin resistance increased from 4.8% to 20.5% [2]. In a study from Poland primary resistance against clarithromycin was seen in 19–21% of the isolates [8]. A study from Pakistan showed a large amount of resistant strains: 89% for metronidazole, 36% for clarithromycin, 37% for amoxicillin, 18.5% for ofloxacin, and 12% for tetracycline. Furthermore, clarithromycin resistance rose from 2005 to 2008 (32% versus 38%, *P* = 0.004) [9]. In a paper from Iran the prevalence of resistance to clarithromycin, metronidazole, erythromycin, amoxicillin, ciprofloxacin, rifampin, nitrofurantoin, and tetracycline was 14.3%, 76.8%, 26.0%, 28.6%, 33.0%, 28.6%, 11.6%, and 18.7%, respectively [10]. On the other hand, in a study from Malaysia no resistance against clarithromycin was reported. This possibly reflects the very low use of macrolides in the general population [11]. 

It is well known that bacterial resistance is the result of selection due to antibiotic pressure. A recent prospective study addressed the resistance to antibiotics in *H. pylori* infection in a study across Europe. The link between outpatient antibiotic use and resistance levels in different countries was studied. Of 2204 patients included, *H. pylori* resistance rates to clarithromycin for adults were 17.5%, 14.1% for levofloxacin, and 34.9% for metronidazole. These rates were significantly higher for clarithromycin and levofloxacin in Western/Central and Southern Europe (>20%) than in Northern European countries (<10%). A significant association was found between the use of long-acting macrolides and clarithromycin resistance (*P* = 0.036). The knowledge of outpatient antibiotic consumption may provide a simple tool to predict the susceptibility of *H. pylori* to antibiotics and to adapt the treatment strategies [12].

In a study from Belgium no resistance to amoxicillin was observed, and tetracycline resistance was very rare (<0.01%). Primary metronidazole resistance remained stable over the years, with significantly lower rates for isolates from children (23.4%) than for isolates from adults (30.6%). Ciprofloxacin resistance increased significantly over the last years in isolates from adults. Primary clarithromycin resistance increased significantly, reaching peaks in 2000 for children (16.9%) and in 2003 for adults (23.7%). A subsequent decrease of the resistance rate down to 10% in both groups corresponded to a parallel decrease in macrolide consumption during the same period [13]. In accordance with this paper, Figure 2 shows a gradual increase in bacterial susceptibility in the course of the study period especially for clarithromycin. It occurred in particular in patients of Turkish descent. Possibly the restrictive policy in use of antibiotics in general in The Netherlands is an explanation for this. Antibiotics are prescription drugs in The Netherlands. General practitioners and specialists only prescribe these drugs if there is an established clinical reason. In many other countries antibiotics can be bought over the counter. Hence, it can be assumed that antibiotic-induced selection pressure is higher in these countries. Why resistance to clarithromycin in patients of Turkish descent is decreasing while it slightly increases in native Dutch patients is not obvious. Possibly this is due to the lower use of macrolides in the Turkish population in the recent years. Decreasing resistance also is reported in other countries [8]. 

Performing culture and antimicrobial susceptibility of the *H. pylori* isolates is a more successful and cost-effective strategy than empirical 10-day treatment in populations with high rates of resistance to clarithromycin [6]. Each previous course of antibiotics is associated with an increase in the risk of antibiotic resistance to the used agent (clarithromycin: RR = 1.5 (*P* = 0.12); metronidazole RR = 1.6 (*P* = 0.002); quinolone RR = 1.8 (*P* = 0.01)) [14]. Clinicians should take the antibiotic history into account before prescribing metronidazole, clarithromycin, or levofloxacin for *H. pylori*. 

A high rate of resistance (49.5 to 72.7%) to amoxicillin was observed in *H. pylori* after two or three unsuccessful eradication attempts. Unsuccessful eradication regimens significantly increase resistance not only to clarithromycin and metronidazole but also to amoxicillin [15]. Megraud advises several approaches: one is to test for clarithromycin resistance so that the triple clarithromycin-based regimen is given only to those who will benefit; a second is to prescribe the drugs sequentially, starting with amoxicillin and a PPI followed by clarithromycin and metronidazole, again with a PPI or the four drugs prescribed concomitantly; a third alternative is to use bismuth-based quadruple therapy, PPI, plus a standardized three-in-one capsule, bismuth subcitrate potassium, metronidazole, and tetracycline [16].

From the present study it can be concluded that resistance of *H. pylori* against metronidazole and clarithromycin stayed rather constant in consecutive years. But, there are differences in presence of resistant strains when native Dutch patients and patients of Turkish descent are compared. Continuous surveillance of antibiotic resistance in *H. pylori* is essential in order to preserve the highest eradication rates. In addition, ethnicity should be taken into account before prescribing antibiotics. 

## Figures and Tables

**Table 1:** Endoscopic diagnoses in 779 patients with positive culture for *H*. *pylori *with known endoscopic diagnosis.

No macroscopic abnormalities	225
Endoscopic signs of gastritis	326
Erosive	67
Nodular	84
Reflux oesophagitis	115
Hiatal hernia	218
Duodenal ulcer	70
Bulbitis	105
Gastric ulcer	27
Cancer	6
Miscellaneous	30

More than diagnosis is possible in a patient.

**Table 2:** Resistant and susceptible strains.

Number	Metronidazole	Clarithromycin
172	?	?
83	R	S
46	R	R
461	S	S
92	S	R

R: resistant; S: sensitive. Unknown means that resistance testing was not successful for this specific antibiotic.

**Table 3:** Antimicrobial resistance in the eight consecutive years in Dutch patients and patients from Turkish descent.

	Dutch/Turkish number	Resistance to clarithromycin	Resistance to metronidazole
2004	38/23	1/7	4/4
2005	99/45	16/8	15/8
2006	98/57	14/9	19/4
2007	117/57	22/8	21/9
2008	87/31	14/5	7/4
2009	84/44	8/11	10/9
2010	49/34	7/4	3/5
2011	44/18	9/1	11/4
		*P* = 0.01	*P* = ns
